# IPMK and β-catenin mediate PLC-β1-dependent signaling in myogenic differentiation

**DOI:** 10.18632/oncotarget.11527

**Published:** 2016-08-23

**Authors:** Giulia Ramazzotti, Anna Maria Billi, Lucia Manzoli, Cristina Mazzetti, Alessandra Ruggeri, Christophe Erneux, Seyun Kim, Pann-Ghill Suh, Lucio Cocco, Irene Faenza

**Affiliations:** ^1^ Cellular Signalling Laboratory, Department of Biomedical and Neuromotor Sciences, University of Bologna, Bologna, Italy; ^2^ Interdisciplinary Research Institute (IRIBHM), Université Libre de Bruxelles, Campus Erasme, Brussels, Belgium; ^3^ Department of Biological Sciences, School of Life Sciences, Ulsan National Institute of Science and Technology, Ulsan, Republic of Korea; ^4^ Department of Biological Sciences, KAIST, Daejeon, Republic of Korea

**Keywords:** myogenic differentiation, phospholipase C-β1, IPMK, β-catenin, inositol phosphates

## Abstract

In previous studies, we have reported that phospholipase C (PLC)-β1 plays a crucial role in myogenic differentiation and we determined the importance of its catalytic activity for the initiation of this process. Here we define the effectors that take part to its signaling pathway. We show that the Inositol Polyphosphate Multikinase (IPMK) is able to promote myogenic differentiation since its overexpression determines the up-regulation of several myogenic markers. Moreover, we demonstrate that IPMK activates the same cyclin D3 promoter region targeted by PLC-β1 and that IPMK-induced promoter activation relies upon c-jun binding to the promoter, as we have shown previously for PLC-β1. Furthermore, our data shows that IPMK overexpression causes an increase in β-catenin translocation and accumulation to the nuclei of differentiating myoblasts resulting in higher MyoD activation. Finally, we describe that PLC-β1 overexpression determines too an increase in β-catenin translocation and that PLC-β1, IPMK and β-catenin are mediators of the same signaling pathway since their overexpression results in cyclin D3 and myosin heavy chain (MYH) induction.

## INTRODUCTION

Myogenic differentiation is a well-defined and highly ordered process that involves several distinct and complex events: myoblasts proliferation, cell-cycle withdrawal, cell migration, alignment and fusion into multinucleated myotubes [1]. The regulation of these processes requires the activation of several transcription factors belonging to two main families: the MRFs (Myogenic Regulatory Factors) and MEF2 (Myocyte Enhancer Factor-2) family [2]. In particular, the MRFs comprise Myf5, MyoD, myogenin and MRF4; they can regulate each other's expression and interact with the MEF2 transcription factors to induce the transcription of muscle-specific genes [3]. The expression of MRFs can drive both myogenic and non-myogenic cell lines into myogenic differentiation [4, 5]. Myf5 and MyoD are expressed early in cell committed to differentiation while myogenin and MRF4 are expressed when cells enter terminal differentiation [6–8].

The fate of the myoblast that is whether it should continue to proliferate or start differentiating, is the result of the balance between positive and negative cell-cycle regulators. Eventually, the activation of differentiation process leads to the expression of late differentiation markers such as troponin, tropomyosin and myosin heavy and light chains that are among the downstream targets of MRFs and MEF2 family.

In previous studies, we investigated the role of phospholipase C(PLC)-β1-dependent signaling in myogenic differentiation using C2C12 murine myoblasts. C2C12 cells can differentiate into myotubes, under low serum conditions and represent a well-established and reproducible model of myogenesis used for studying many muscle-specific genes and proteins [9].

PLC-β1 belongs to the family of PLCs that comprises 13 enzymes, divided into six classes based on their different domain composition. PLC-β1 catalyzes the hydrolysis of the signaling lipid phosphatidylinositol 4,5-bisphosphate (PI(4,5P)_2_) generating two important second messengers: inositol 1,4,5,-trisphosphate (IP_3_) and diacylglycerol (DAG), which in turn regulate several cellular processes [10]. PLC-β1 presents two splicing variants: β1a and β1b that differ in their C-terminal region. The two variants show different cellular distribution: while PLC-β1a is located specially in the cytosol, PLC-β1b is mainly distributed to the nucleus [11].

We have previously demonstrated that during C2C12 myoblast differentiation PLC-β1 expression is highly induced and that its catalytic activity is essential for the onset of the differentiation program. Moreover, the activation of PLC-β1-dependent pathway determines the up-regulation of cyclin D3 promoter activity leading to an increase in cyclin D3 expression during myogenic differentiation. In a following study, we identified the cyclin D3 promoter region specifically targeted by PLC-β1 signaling activity, and we described the transcription factor c-jun as the mediator for promoter activation [12–15].

In this study, we wanted to understand better the pathway induced by PLC-β1 during myogenic differentiation. We hypothesized that the second messenger IP_3_ generated by PLC-β1 activity could be the substrate of Inositol Polyphosphate Multikinase (IPMK) leading to the production of higher phosphorylated inositol species.

IPMK is a multifunctional enzyme that shows both IP3- and IP4-kinase, and PI3-kinase activity. It is essential for the production of IP_4_ (both Ins(1,3,4,5)P_4_ and Ins(1,4,5,6)P_4_) and IP_5_ [16, 17]. IPMK is crucial for the production of higher phosphorylated inositol species, since it is the only enzyme capable to produce Ins(1,4,5,6)P_4_ and IP_5_. Therefore IPMK depletion results in the nearly complete elimination of intracellular IP_6_ and IP_7_ [17]. IPMK can also act as a PI3-kinase phosphorylating PIP_2_ at the 3, position in order to produce PIP_3_ [18]. Moreover, it shows catalytic-independent activities as it can bind and stabilize the mTOR (mammalian Target of Rapamicin) complex [19] as well as function as a transcriptional co-activator for serum response factor [19], CREB (cAMP response element)-binding protein [20] and p53 [21,22].

Since it was described in a different model that IP_5_ produced by IPMK could induce the Wnt/β-catenin pathway, we hypothesized that β-catenin could be a mediator of PLC-β1 pathway in myogenic differentiation [23]. The activation of Wnt/β-catenin pathway leads to the accumulation of β-catenin and its translocation to the nucleus where it induces downstream gene expression. When the pathway is not activated, β-catenin is bound to axin, adenomatous polyposis coli (APC), glycogen synthase kinase (GSK) 3β and casein kinase 1 that induce β-catenin proteasomal degradation [24]. Βeta-catenin target genes are involved in several cellular processes, such as proliferation, differentiation, survival and angiogenesis, as well as cancer initiation and progression [25].

Our results show that IPMK promotes myogenic differentiation of C2C12 cells. Moreover, IPMK is involved in PLC-β1-dependent signaling since it activates the same cyclin D3 promoter region targeted by PLC-β1, and we show that the promoter activation depends upon c-jun binding. Furthermore, we investigated the involvement of β-catenin in myogenic differentiation and we present its nuclear translocation in response to IPMK overexpression. Taken together our data is useful to define better the PLC-β1-dependent signaling pathway in myogenic differentiation.

## RESULTS

### IPMK overexpression promotes myogenic differentiation

Since we demonstrated that PLC-β1-depedent signaling is involved in myogenic differentiation, we wanted to understand further how this process is accomplished [13]. PLC-β1 activation yields to diacylglycerol (DAG) and inositol (1,4,5)- trisphosphate (IP_3_) production, therefore we hypothesized that IP_3_ could be the substrate of Inositol Polyphosphate Multikinase (IPMK) generating higher phosphorylated inositol species.

**Figure 1:**
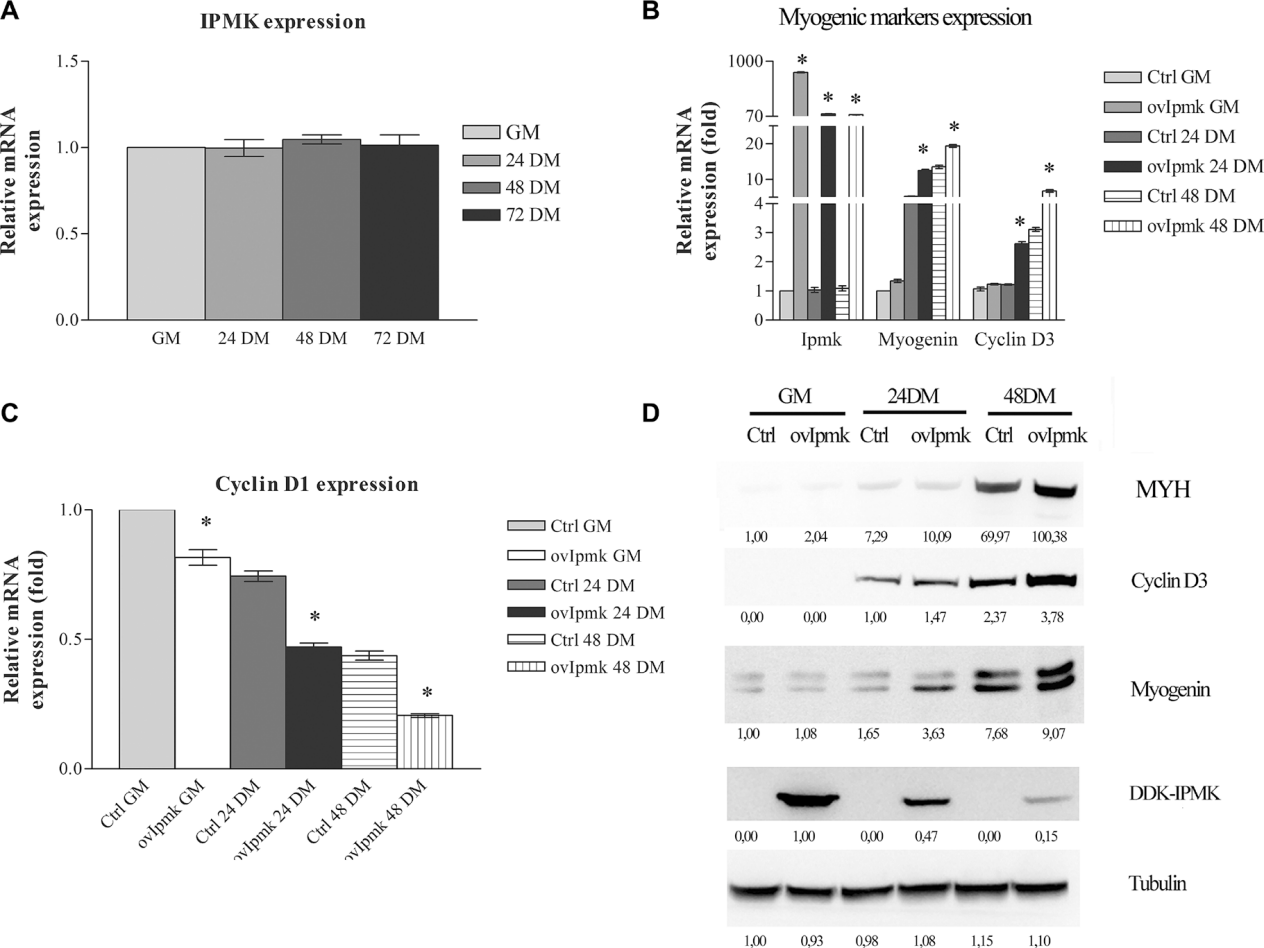
IPMK overexpression promotes myogenic differentiation (**A**): IPMK expression was evaluated by real-time PCR in C2C12 cells in growing medium (GM) and after 24, 48 and 72 h culture in differentiation medium (respectively 24 DM, 48 DM, 72 DM). (**B**), (**C**) and (**D**): IPMK was overexpressed in C2C12 cells and the expression of myogenic markers was evaluated in growing cells (GM), 24 h, and 48 h after switching to differentiation medium (24 DM and 48 DM). The expression of IPMK, myogenin, cyclin D3 (panel B) and cyclin D1 (panel C) was evaluated by real –time PCR. Data are from three independent experiments, *p < 0.05 vs corresponding ctrl sample. The expression of myosin heavy chain (MYH), cyclin D3, myogenin, and DDK-tagged IPMK was evaluated by Western blot using tubulin as loading control (panel D). Data are from three independent sets of experiments.

First, we evaluated IPMK expression during the differentiation of C2C12 cells. Since we have not found a good commercial antibody for IPMK, we tested its expression in real-time PCR. Figure 1A shows IMPK expression in growing (GM) and differentiating cells, respectively 24 h, 48 h and 72 h after medium switch (DM). It is clear that IPMK expression did not vary during myogenic differentiation.

Then, we overexpressed IPMK in C2C12 cells, we induced the differentiation and we tested at different time points the expression of genes involved in the differentiation process, namely: myogenin, cyclin D3, and cyclin D1. Samples were collected following the incubation in growth medium (GM) and 24 h, and 48 h after differentiation induction (DM). As shown in Figure 1B IPMK overexpression determined an increase in the expression of myogenin and cyclin D3 even before the switch from growth medium (GM) to differentiation medium (DM). It caused also a marked decrease in cyclin D1 expression (Figure 1C), strongly supporting a role for IPMK in myogenic differentiation.

Moreover, we tested if the changes that we observed at the transcriptional level corresponded to changes in protein expression. Therefore, we evaluated myogenin, cyclin D3 and, myosin heavy chain (MYH) expression in whole cell lysates of C2C12 cells overexpressing DDK-tagged IPMK, kept in GM and 24, and 48 hours after DM administration. As displayed in Figure 1D, cells overexpressing IPMK showed higher levels of differentiation markers.

The above data strongly suggest that IPMK plays a role in promoting the differentiation of C2C12 skeletal muscle cells.

### Effects of IPMK on cyclin D3 promoter activation

In a previous study, we have demonstrated that PLC-β1-dependent signaling determined cyclin D3 promoter activation during muscle differentiation and it targeted a specific promoter region, i.e. the region spanning from -446 to -190 bp, containing the binding site for the transcription factor c-*jun* [12].

**Figure 2:**
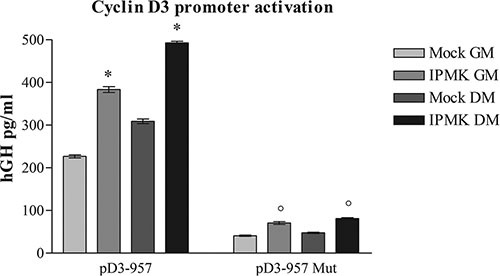
Effects of IPMK on cyclin D3 promoter activation C2C12 cells were co-transfected with an empty vector (mock) or a vector coding for IPMK (IPMK) and either with a reporter vector coding for human growth hormone (hGH) under control of a fragment of cyclin D3 promoter (pD3-957) or with the same vector bearing a mutation in c-jun consensus sequence in the promoter region (pD3-957 mut). hGH production was evaluated in growing cells (GM) and 24 h after the induction of differentiation (DM). Data are from three independent experiments, *p < 0.05 vs corresponding mock sample and °*p* < 0.05 vs corresponding pD3-957 sample.

In order to further assess IPMK involvement in myogenic differentiation, we investigated the ability of IPMK to activate the same cyclin D3 promoter region targeted by PLC-β1. Briefly, C2C12 cells were transfected with pD3-957 reporter vector, coding for the human growth hormone (hGH) under control of the −957 to +1 cyclin D3 promoter fragment and with an empty vector (mock) or a vector coding for IPMK. HGH production was evaluated 24 h after differentiation induction. Notably, Figure 2 shows that transient overexpression of IPMK caused an increase in hGH production due to higher cyclin D3 promoter activity and the activity was even higher when GM was replaced with DM.

Moreover, we performed the reporter gene assay using a pD3-957 vector in which c-*jun* binding site has been mutated (pD3-957mut). As described in [13], PLC-β1-dependent cyclin D3 promoter activation requires c-*jun* binding. In order to understand if IPMK takes part to the same pathway induced by PLC-β1 during muscle differentiation, we tested IPMK ability to activate the mutated cyclin D3 promoter. We co-transfected C2C12 cells with an empty vector (mock) or with IPMK coding vector and with wild type (pD3-957) or mutated pD3-957 (pD3-957mut) vector. Then we compared hGH production 24 h after the differentiation induction. Figure 2 shows that IPMK overexpression could no longer activate the mutated promoter even after DM administration, while cells transfected with native pD3-957 vector showed the expected hGH production. Therefore, these data underline that IPMK takes part to PLC-β1-dependent pathway, as it requires the same transcription factor, i.e. c-*jun*, in order to activate cyclin D3 promoter.

### IPMK promotes nuclear β-catenin translocation during myogenic differentiation

**Figure 3:**
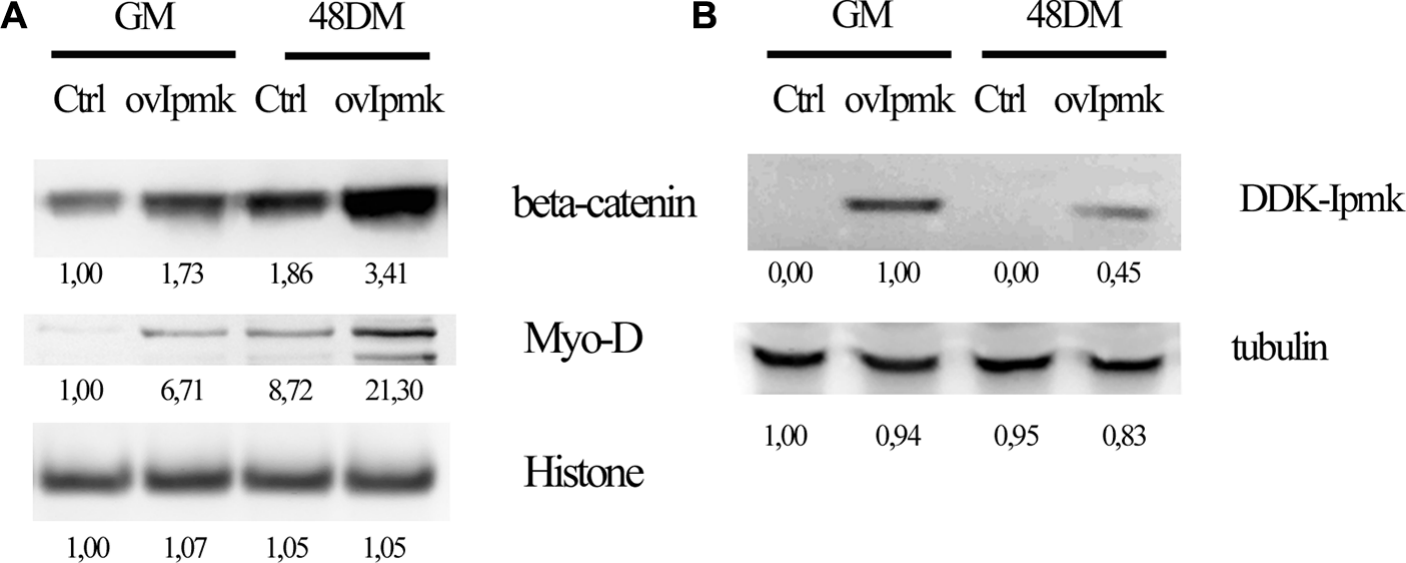
IPMK promotes β-catenin nuclear accumulation C2C12 cell were transfected with an empty vector (Ctrl) or DDK-tagged IPMK vector (ovIpmk). Cells were grown in growing medium (GM) or in differentiation medium (48 DM) for 48 h and (**A**) nuclear extracts were tested for β-catenin and Myo-D expression. Histone was used as loading control. (**B**) IPMK expression was tested on whole cell lysate from the same samples. Data are from three independent experiments.

It has been described that canonical Wnt signaling is involved in myoblast differentiation. Wnt signaling induces β-catenin shuttling from the cytoplasm to the nucleus, but it is still unknown the mechanisms that lead to β-catenin activation [26]. It was also demonstrated that inositol pentakisphosphate (IP_5_) can stimulate casein kinase2 (CK2) activity promoting nuclear β-catenin accumulation [27]. Therefore, we explored whether IPMK could be in involved in regulating β-catenin accumulation in the nuclear compartment during myogenic differentiation. We overexpressed DDK-tagged IPMK in C2C12 cells and we probed the nuclear lysates of growing and differentiating cells, 48 h after switching to DM. Firstly, these results indicated that during myogenic differentiation nuclear β-catenin accumulation is increased. Moreover, nuclear extracts of samples that overexpressed IPMK presented a marked raise in β-catenin as well as in MyoD, a well-known myogenic marker, accumulation compared to controls (Figure 3A). IPMK overexpression was checked in whole cell lysates in growing (GM) and differentiating (48 DM) samples (Figure 3B).

Therefore, IPMK overexpression induces β-catenin accumulation to the nuclear compartment and it promotes myogenic differentiation, as shown by MyoD increase, a fundamental event in the differentiation process.

### Defining PLC-β1 signaling in myogenic differentiation: IPMK, β-catenin and cyclin D3

Since we demonstrated that β-catenin is a downstream effector of IPMK activity, we wanted to investigate the effect of PLC-β1 overexpression on β-catenin nuclear accumulation.

**Figure 4:**
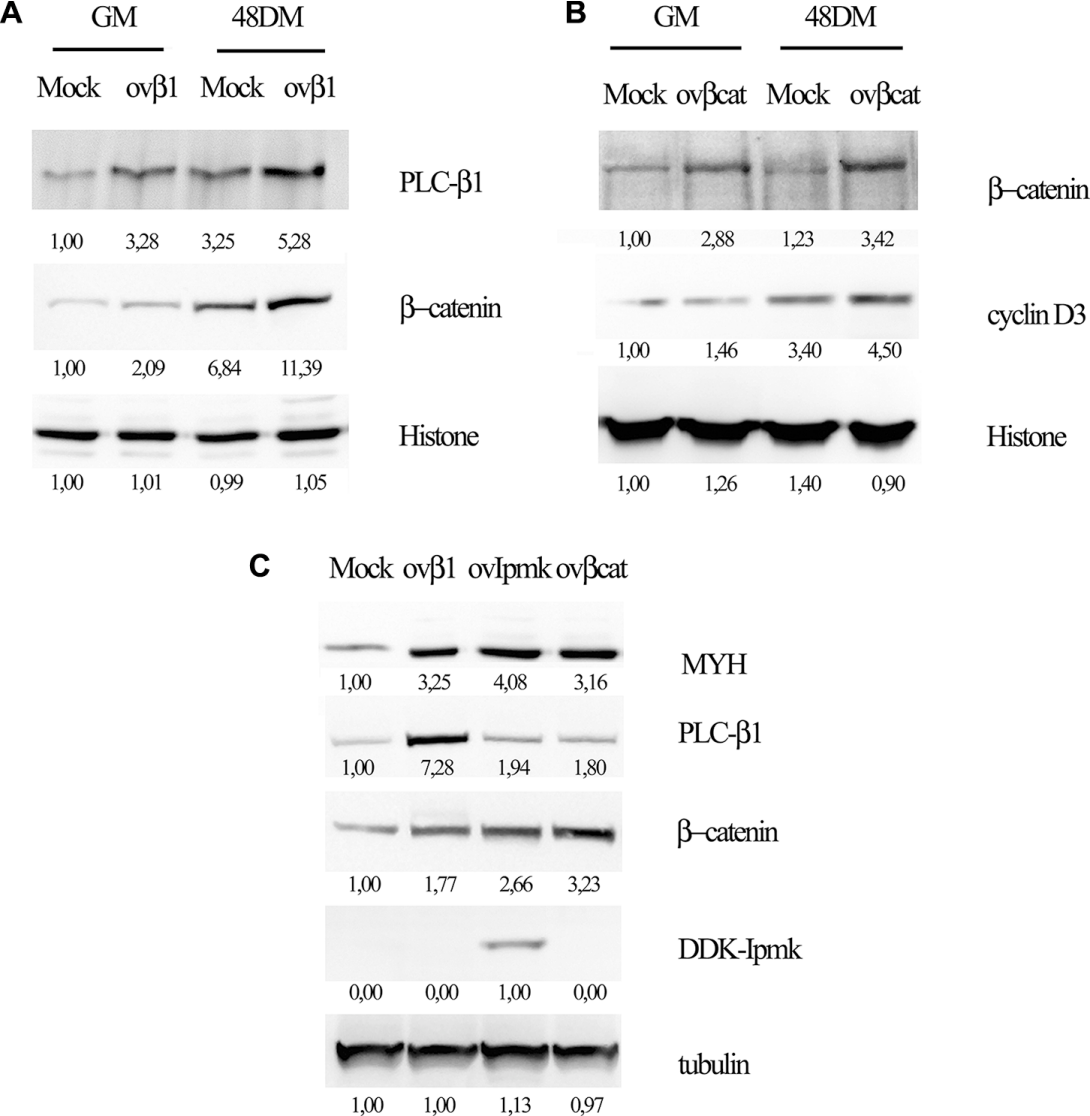
Effects of PLC-β1, β-catenin and IPMK overexpression on myogenic differentiation C2C12 cells were transfected with and empty vector (Mock) or a vector coding for PLC-β1 (ov-β1, panel **A** and **C**) or for β-catenin (ovβcat, panel **B** and C) or DDK-tagged IPMK (ovIMPK, panel C). After 48 h of differentiation, growing cells (GM) and differentiating cells (48 DM) were harvested. Nuclear extracts were tested for β-catenin (panel A) and cyclin D3 (panel B) expression and histone was used as loading control. Whole cell lysate (panel C) were tested for myosin heavy chain expression (MYH) and tubulin was used as loading control. Data are from three independent experiments.

We transfected C2C12 cells with a vector coding for PLC-β1 and we induced the differentiation, then we tested nuclear extracts for β-catenin expression. As shown in Figure 4A PLC-β1 overexpression determined higher β-catenin nuclear accumulation as compared to mock-transfected sample. Hence, as we showed for IPMK also PLC-β1 overexpression induced β-catenin accumulation to the nuclear compartment.

Moreover, we investigated the effect of β-catenin overexpression on cyclin D3 in order to define the factors involved in PLC-β1 pathway during myogenesis. We overexpressed β-catenin in C2C12 myoblasts and we tested the nuclear extracts for cyclin D3 expression 48 h after inducing the differentiation. Figure 4B shows that β-catenin overexpression determines cyclin D3 accumulation both in growing and in differentiating cells.

Furthermore, we investigated the effect of the overexpression of PLC-β1, β-catenin and IPMK on myogenic differentiation. Cells were transfected with the vectors coding for the proteins cited above and whole cell lysates were tested for myosin heavy chain (MYH) expression 48 h after the induction of differentiation. Figure 4C displays that the overexpression of all these proteins determined an increase in myosin heavy chain expression as compared to the mock-transfected sample, confirming that they are all involved in promoting myogenic differentiation.

Finally, in order to determine whether the effect of IPMK overexpression was due to its catalytic activity, we overexpressed the following IPMK mutants: a kinase dead mutant (IPMK SA), an inositol-binding site mutant (IPMK KA) and a double mutant (IPMK KA/SA). 24 hours after transfection growth medium was replaced with differentiation medium and we evaluated the expression of myogenic markers 24 h and 72 h after switching the medium.

**Figure 5:**
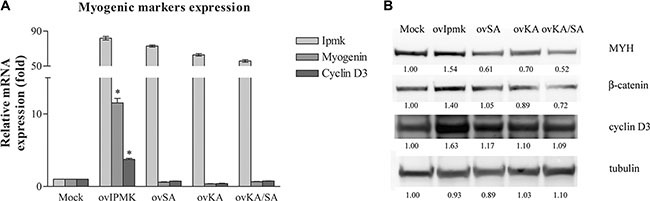
Effects of IPMK mutants' overexpression on myogenic differentiation C2C12 cell were transfected with an empty vector (Mock) or vectors coding for wild type IPMK (ovIpmk), IPMK kinase-dead mutant (ovSA), IPMK mutated in the inositol-binding site (ovKA) or IPMK double mutant (ovKA/SA). 24 h after transfection growth medium was switched to differentiation medium (DM). (**A**) 24 h after changing the medium to DM, IPMK, myogenin and cyclin D3 expression was evaluated by real –time PCR. Data are from three independent experiments, *p < 0.05 vs mock sample. (**B**) 72 h after changing the medium to DM, whole cell lysates were tested for myosin heavy chain, β-catenin and cyclin D3 expression. Tubulin was used as loading control.

24 h after the induction of differentiation, we compared by real-time PCR the expression of myogenin and cyclin D3 between cells transfected with an empty vector and with vectors coding for wild type IPMK and for IPMK mutants. Figure 5A shows that the overexpression of IPMK mutants does not determine a variation in myogenic markers expression compared to the mock- transfected control sample. 72 hours after switching to differentiation medium, we checked myosin heavy chain, β-catenin and cyclin D3 expression on whole cell lysates of transfected cells. Figure 5B shows that while the overexpression of wild type IPMK (ovIPMK) determines an increase in markers expression, the expression of the three mutants (ovSA, ovKA, ovKA/SA) does not induce myogenic markers.

## DISCUSSION

Skeletal muscle differentiation is an extremely complicated and highly regulated process that involves cellular interactions and extracellular signaling molecules, including Wnts, Sonic hedgehog (Shh), and bone morphogenetic proteins (BMPs), which are secreted from the surrounding environment. These signaling proteins induce transcription factor networks that cause several cell events resulting in cell differentiation [28, 29].

In previous studies, we have described the relevance of PLC-β1-dependent signaling pathway in myogenic differentiation since not only PLC-β1 expression increases during differentiation [14] but its overexpression is able to induce the differentiation process. Moreover, PLC-β1 silencing results in the inhibition of the onset of the differentiation program [12]. Muscle cells differentiation is characterized by the induction of myogenic markers and by the increase of cyclin D3 expression due to transcriptional activation of its promoter. We demonstrated that PLC-β1-dependent signaling pathway can activate cyclin D3 promoter during the differentiation of myoblasts to myotubes, indicating that PLC-β1 is an important regulator of cyclin D3 gene expression [12, 14, 30]. In this study, we aimed to elucidate further PLC-β1 signaling pathway and its downstream targets in myogenic differentiation. The data we presented indicate that IPMK and β-catenin are both involved in determining the induction of myogenin, cyclin D3 and myosin heavy chain (MYH) during the differentiation process.

Despite the importance of higher phosphorylated inositol species for several cellular functions, IPMK physiologic and pathologic regulations are still largely unexplored. Recently, a group has reported that IPMK expression is profoundly reduced in Huntington's disease. They showed that IPMK depletion causes neural dysfunction, and restoration of IPMK delays the progression of behavioral abnormalities and rescues striatal pathology [31]. Until now, studies about the role of IPMK in skeletal muscle differentiation or muscular diseases were lacking. This prompted us to explore IPMK involvement in myogenic differentiation. Since we showed that PLC-β1 catalytic activity is required for myogenic differentiation [13], we hypothesized that the second messenger IP_3_, produced by PLC-β1, could be the substrate of IPMK to generate higher phosphorylated inositol species that could be required for the differentiation process. Firstly, we investigated whether IPMK expression showed any changes during C2C12 differentiation. However, IPMK expression did not show any relevant variation. Since IPMK silencing resulted in cell death, probably due to inositol pentakisphosphate (IP_5_) depletion, we decided to evaluate the effects of its overexpression on several genes involved in myogenic differentiation. Our data showed that IPMK overexpression promoted the differentiation of C2C12 skeletal muscle cells as it elicited the induction of myogenin, cyclin D3 and myosin heavy chain.

In previous studies, we have shown that PLC-β1 is able to induce the differentiation of C2C12 myoblasts by targeting a specific region of the cyclin D3 promoter and that PLC-β1 is a key regulator of cyclin D3 transcription [12]. Our data showed that the increase in cyclin D3 expression is actually due to the activation of the transcription factor *c-jun*, a member of AP-1 transcription complex, through PLC-β1-dependent signaling pathway [13]. In this work, we show that IPMK is also involved in the regulation of cyclin D3 expression. In order to identify the transcription factor activated by IPMK, we mutated *c-jun* binding site in a specific cyclin D3 promoter region that presents the binding sites for several other transcription factors. The failure of IPMK overexpression to activate the mutated promoter confirms the crucial role of *c-jun* in IPMK-dependent promoter activation. Therefore, IPMK overexpression determines an increase in cyclin D3 promoter activation by targeting the same transcription factor activated by PLC-β1 overexpression, i.e. *c-jun,* suggesting that the two enzymes take part to the same signaling pathway.

It has been demonstrated that inositol 1,3,4,5-tetrakisphosphate (IP_4_), inositol pentakisphosphate (IP_5_) and inositol hexakisphosphate (IP_6_) can stimulate casein kinase 2 (CK2) activity which in turn suppresses β-catenin degradation and binding to adenomatous polyposis coli (APC) promoting β-catenin accumulation to the nuclear compartment [23, 32–34]. Once in the nucleus β-catenin recruits cofactors of the TCF/LEF family and induces the transcription of its target genes [35]. Consequently, inositol polyphosphates can positively regulate the Wnt/ β-catenin pathway. Wnt signaling pathway plays an important role in muscle development and regeneration as it is involved in the switching from cell proliferation to myogenic differentiation in mouse myoblasts [26, 36].Therefore, we decided to analyze the expression of the main component of the canonical Wnt signaling pathway, i.e. β-catenin, in response to IPMK overexpression. Beta-catenin plays an important role in a wide variety of cellular processes, including stem cell proliferation and tissue regeneration, as well as in cancer progression [37]. We demonstrated that IPMK overexpression is able to induce β-catenin accumulation to the nuclear compartment, which is a fundamental event in myogenic differentiation progression. Moreover, it has been proven that *c-jun* is one of β-catenin target genes since its promoter is activated by β-catenin overexpression [38], supporting the hypothesis that β-catenin might act downstream IPMK in order to activate cyclin D3 promoter.

Furthermore, in order to determine whether PLC-β1, IPMK, β- catenin and cyclin D3 belong to the same signaling pathway activated during myogenic differentiation, we performed a series of overexpression experiments. We showed that PLC-β1 overexpression induces β-catenin accumulation in the nucleus and in turn, β-catenin overexpression determines an increase in cyclin D3 but it does not affect PLC-β1 expression. Moreover, both PLC-β1 and IPMK overexpression causes β-catenin accumulation, as shown in Figure 4C. Additionally, the expression levels of myosin heavy chain increased considerably following to the overexpression of PLC-β1, β-catenin or IPMK.

Finally, Xu et al. demonstrated that IPMK is able to induce the transcription of several genes independently of its inositol phosphate and lipid kinase activities. In particular, they showed that IPMK can induce several immediate early genes in neural activation [20], and can stimulate p53-dependent transcription in stress responses by binding to p53 and enhancing its acetylation by p300 acetyltransferase [21]. Therefore, we tested whether IPMK catalytic activity was required for myogenic markers induction. The overexpression of three IPMK mutants, respectively a kinase dead-mutant, an inositol binding site-mutant and a double mutant, failed to induce myogenic markers expression demonstrating that IPMK lipid kinase activity is required for myoblasts differentiation.

Taken together our data identify new mediators of PLC-β1- dependent signaling pathway in myogenic differentiation. The model we propose suggests that following to PLC-β1 accumulation PIP_2_ is hydrolyzed producing IP_3_, which is sequentially phosphorylated by IPMK leading to the formation of higher phosphorylated inositol species, like IP_4_, IP_5_ and IP_6_. IP_5_ accumulation determines β-catenin translocation to the nucleus where it activates c-jun, which in turn activates cyclin D3 promoter. Although further studies are required to elucidate all the molecular mechanisms underlying PLC-β1 signaling in myogenic differentiation, it is striking to state that these results identify a series of events related to a common pathway that lead to the same target activation, that is cyclin D3 promoter.

## MATERIALS AND METHODS

### Cell culture, differentiation and transfection

Murine myoblast C2C12 cells (American Type Culture Collection, Manassas, VA) were cultured at sub-confluent density in growth medium (GM) consisting of high glucose DMEM (Sigma–Aldrich, St. Louis, MO) supplemented with 10% fetal bovine serum (Sigma-Aldrich) and antibiotics (100 U/ml penicillin, 100 mg/ml streptomycin, Sigma–Aldrich). To induce differentiation, confluent C2C12 cells were cultured in differentiation medium (DM) consisting of DMEM supplemented with 2% horse serum (Sigma-Aldrich) and antibiotics, for 24, 48 or 72 h as reported in the Figures.

For protein expression analysis and reporter gene assays, C2C12 cells were transfected using Lipofectamine 2000 from Life Technologies, following manufacturer's instructions. One day before transfection 1,5 × 10^5^ cells per well were seeded in 6-well plates or 1,5 × 10^6^ cells in T25 flasks, 24 h post-transfection the medium was replaced either with growing medium (GM) or differentiation medium (DM), and the cells were kept in this medium for the time described in the Figures.

### Expression vectors

The vector coding for Myc-DDK- tagged mouse IPMK (TrueORF clone, MR206205) was purchased from OriGene (Rockville, MD).

The pD3-957 plasmid containing the human growth hormone (hGH) reporter gene under the control of cyclin D3 promoter region spanning from−957 bp to the transcription start site was generated as described in [39]. The *c-jun* mutated vector (pD3-957mut) was obtained by sited-directed mutagenesis in pD3-957 promoter fragment as reported in [13].

For PLC-β1 expression experiments, PLC-β1a was cloned in pcDNA/2.1 vector (LifeTechnologies, Carlsbad, CA).

pcDNA3.1/nV5-DEST-β-catenin expression vector was purchased from Addgene (Cambridge, MA), kindly provided by Valeri Vasioukhin, Fred Hutchinson Cancer Research Center, Seattle.

pCMV vectors coding for human wild type IPMK, IPMK kinase-dead mutant (IPMK SA), IPMK mutated in the inositol-binding site (IPMK KA) and IPMK double mutant (IPMK KA/SA) were designed as described in [40].

### Reporter gene assay

For each sample 1,5 × 10^5^ C2C12 cells were co-transfected using Lipofectamine 2000, as described above, with 1 μg of expression vector and 1 μg of reporter vector. We used empty pcDNA 2.1 (Mock) and TrueORF- Ipmk as expression vectors. pD3-957 or its *c-jun*-mutated construct (pD3-957mut) were used as reporter vectors. 24 h after transfection medium was replaced either with growth medium (GM) or differentiation medium (DM). The promoter activity was evaluated 24 h after the medium switch by measuring hGH concentration in cell medium by using hGH ELISA kit (Roche, Indianapolis, IN).

### RNA extraction, retrotranscription and real-time PCR

Total RNA was extracted form growing or differentiating C2C12 cells at different time points, using RNeasy mini kit (Qiagen, Hilden, Germany). RNA was quantified using a Nanodrop and cDNAwas synthesized starting from 2 μg of total RNA using 200 U of M-MLV retro-transcriptase (Promega, Madison,WI), 0.5 μg of oligo-dT primers, 25 U ribonuclease inhibitor, 10 mM of each dNTP. The reactions were incubated for 1 h at 42°C.

Gene expression was determined by using a TaqMan based real-time PCR method. We used the following validated assays: Ipmk Mm01148668_m1, myogenin Mm00446194_m1, cyclin D3 Mm01273583_m1, cyclin D1 Mm00432359_m1 (Applied Biosystems, Foster City, CA). GAPDH was used as the reference housekeeping gene (assay no. Mm99999915_g1, Applied Biosystems). Quantitative RT-PCR reactions were performed using TaqMan PCR universal master mix using the ABI PRISM 7300 real-time PCR machine (Applied Biosystems). Samples were analyzed in triplicate. Gene expression was analyzed using relative quantification and the ΔΔCt method. The results of different sets of experiments were statistically analyzed by GraphPad Prism version 3.02.

### Protein extraction and western blot analysis

Whole cell lysate were obtained by lysing the cells in M-PER extraction reagent (Thermo Fisher Scientific) supplemented with Halt protease inhibitor cocktail (Thermo Scientific).

For nuclear purification 2,5 × 10^6^ cells were suspended in 250 μl of swelling buffer [10 mM Tris-HCl (pH 7.4), 1% Nonidet P-40, 10mM β-mercaptoethanol, 0.5mM phenylmethylsulfonyl fluoride] supplemented with Halt protease inhibitor cocktail and kept on ice for 5 minutes. Then 250 μl of ice-cold water were added. After 3 min, cells were sheared by eight passages through a 23-gauge hypodermic needle. Nuclei were recovered by centrifugation at 400 g at 4°C for 6 min and washed once in 400 μl of washing buffer [10 mM Tris-HCl (pH 7.4) and 2 mM MgCl2, plus protease inhibitors as describe above]. The purity of the isolated nuclei was determined by western blotting using an anti-β-tubulin antibody. Protein concentrations were determined by the method of Bradford.

For western blot analysis, 50 μg of proteins from whole-cell extracts or 20 μg from nuclear extracts were separated on 4–12% polyacrylamide-0.1% SDS gels (Life Technologies). Proteins were transferred to nitrocellulose membranes and proved with specific antibodies, detected with horseradish peroxidase-conjugated secondary antibody. The immunoreactive bands were visualized using an ECL enhanced chemiluminescence system (Thermo Scientific) and visualized in a Kodak digital image station 2000R. The expression of specific proteins was analyzed by using the following antibodies: anti-MYH monoclonal antibody (sc-376157), anti-myogenin monoclonal antibody (sc-12732), anti-MyoD monoclonal antibody (sc-377460) and anti-PLC-β1 (sc-9050) antibody were purchased from Santa Cruz Biotechnology (Santa Cruz, CA); monoclonal Cyclin D3 (#2936), anti-β-catenin antibody (#9587) and anti-Histone H2A.X antibody (#2595) from Cell Signaling Technology, Inc., (Danvers, MA). Anti-Flag M5 monoclonal antibody (F1804) used for detecting DKK-tag and anti-βtubulin antibody (T7816) were purchased from Sigma-Aldrich (MO, USA).

### Statistical analysis and image analysis

Real-time PCR results of myogenic markers expression and ELISA results of cyclin D3 promoter activation are reported as the mean ± SD of three independent sets of experiments. Data were analyzed using two-way ANOVA with Tukey's tests for multiple comparisons. *P* values < 0.05 were considered statistically significant. All analyses were performed with GraphPad Prism version 3.02 for Windows.

Images were quantified with ImageJ software. Data are from at least three independent experiments.
